# An Assessment of National Maternal and Child Health Policy-Makers’ Knowledge and Capacity for Evidence-Informed Policy-Making in Nigeria

**DOI:** 10.15171/ijhpm.2016.132

**Published:** 2016-10-08

**Authors:** Chigozie Jesse Uneke, Issiaka Sombie, Namoudou Keita, Virgil Lokossou, Ermel Johnson, Pierre Ongolo-Zogo

**Affiliations:** ^1^Knowledge Translation Platform, African Institute for Health Policy and Health Systems Studies, Ebonyi State University, Abakaliki, Nigeria.; ^2^Organisation Ouest Africaine de la Santé, Bobo-Dioulasso, Burkina Faso.; ^3^Hopital Central Yaounde, Yaoundé, Cameroon.

**Keywords:** Maternal, Newborn, Child Health, Policy-Making, Evidence Use

## Abstract

**Background:** There is increasing interest globally in the use of more rigorous processes to ensure that maternal, newborn, and child health (MNCH) care recommendations are informed by the best available research evidence use. The purpose of this study was to engage Nigerian MNCH policy-makers and other stakeholders to consider issues around research to policy and practice interface and to assess their existing knowledge and capacity on the use of research evidence for policy-making and practice.

**Methods:** The study design is a cross-sectional evaluation of MNCH stakeholders’ knowledge as it pertains different dimensions of research to practice. This was undertaken during a national MNCH stakeholders’ engagement event convened under the auspices of the West African Health Organization (WAHO) and the Federal Ministry of Health (FMoH) in Abuja, Nigeria. A questionnaire was administered to participants, which was designed to assess participants’ knowledge, capacity and organizational process of generation, synthesis and utilization of research evidence in policy-making regarding MNCH.

**Results:** A total of 40 participants signed the informed consent form and completed the questionnaire. The mean ratings (MNRs) of participants’ knowledge of electronic databases and capacity to identify and obtain relevant research evidence from electronic databases ranged from 3.62-3.68 on the scale of 5. The MNRs of participants’ level of understanding of a policy brief, a policy dialogue and the role of researchers in policy-making ranged from 3.50-3.86. The MNRs of participants’ level of understanding of evidence in policy-making context, types and sources of evidence, capacity to identify, select, adapt, and transform relevant evidence into policy ranged from 3.63-4.08. The MNRs of the participants’ organization’s capacity to cover their geographical areas of operation were generally low ranging from 3.32-3.38 in terms of manpower, logistics, facilities, and external support. The lowest MNR of 2.66 was recorded in funding.

**Conclusion:** The outcomes of this study suggest that a stakeholders’ engagement event can serve as an important platform to assess policy-makers’ knowledge and capacity for evidence-informed policy-making and for the promotion of evidence use in the policy process.

## Background

In most countries of the world, including a considerable number of low- and middle-income countries, there is increasing interest in the use of more rigorous processes to ensure that healthcare recommendations are informed by the best available research evidence.^1,2^ This is supported by numerous scientific reports which have indicated that evidence from research can enhance health policy development by identifying new issues for the policy agenda, informing decisions about policy content and direction or by evaluating the impact of policy.^3-6^ The World Health Organization (WHO) in a previous report clearly indicated that better use of research evidence in development policy-making can save lives through more effective policies that respond to scientific and technological advances, use resources more efficiently and better meet citizens’ needs.^7^

In response to this global interest in evidence-to-policy process in the health sector, the Nigeria government recognized the importance of evidence-based health policy as a critical requirement for the improvement of the country’s health systems.^8,9^ As part of the government’s effort to promote and drive evidence-informed policy-making, the Nigeria Evidence-Based Health System Initiative (NEHSI) was established with the aim of building a responsive evidence-based health system, with emphasis on primary healthcare principally to improve maternal and child health outcomes.^10,11^

In a recent systematic review on maternal and child health interventions in Nigeria from 1990-2014, Kana and colleagues^12^ noted that poor maternal and child health indicators have been a recurring public health challenge in Nigeria since documentation of national maternal, newborn, and child health (MNCH) statistics began in the early 1990s. For instance, it is reported that each year in Nigeria, more than a quarter million neonates die, which translates to approximately 700 neonates every day.^13^ A number of previous studies have indicated that low birth weight, lack of antenatal care, maternal illness, mother’s age, prematurity, and birth asphyxia are strongly associated with neonatal mortality in Nigeria.^14,15^

Regarding maternal health, out of 529 000 annual global maternal deaths, an estimated 52 900 Nigerian women die from pregnancy related complications, thus, a woman’s chance of dying from pregnancy and childbirth in the country is 1 in 13.^16^ According to available reports, the main causes of maternal mortality in Nigeria are: haemorrhage (23%), infection (17%), unsafe abortion (11%), obstructed labour (11%) and toxaemia/eclampsia/hypertension (11%), Malaria (11%), anaemia (11%), and others including HIV and AIDS contribute about (5%).^17-19^ Other factors underlying maternal mortality include lack of awareness about complications in pregnancy and on the need to seek medical intervention early; lack of transportation to the health facilities where maternal healthcare can be provided; inability to pay for services, etc.^16,17,19,20^

To address these MNCH challenges in Nigeria, the development and implementation of evidence-informed policies and promotion of initiatives such as NEHSI are very imperative. This is supported by the outcome of the systematic review of Kana and colleagues^12^ who noted that the development of evidence-based MNCH policies, implementation and publication of interventions corresponded with the downward trend of maternal and child mortality in Nigeria. However, despite the introduction of NEHSI^11^ and other similar initiatives such as the Nigeria independent accountability mechanism for MNCH,^21^ there is still insufficient interest and commitment on the part of policy-makers in transfer and uptake of research evidence into the health policy-making process in Nigeria.^22^

One of the most important factors responsible for the lack of sufficient commitment to evidence-to-policy process by Nigerian policy-makers is their capacity constraints to access, synthesize, adapt and utilize available research evidence.^23,24^ According Dawad and Veenstra,^25^ without adequate capacity, in knowledge translation/management and health policy research, policy-makers will not have the capacity to access and synthesize sound information on which to base decisions and the potential for shared learning will be lost. Furthermore, Green and Bennett,^26^ noted that knowledge and skill constraints associated with accessing evidence from various sources and competency in making use of the evidence appropriately are among the most important capacity needs of policy-makers.

However, before designing any capacity enhancement strategy for evidence-to-policy process, there is need to assess the existing knowledge and capacity on the use of research evidence which the policy-makers possess. Deans and Ademokun^27^ had noted in their report that those who seek to build capacity for evidence-informed policy need to understand the actual capacity gaps of policy-makers. Available reports indicate that there are only very few studies that have attempted to evaluate the individual and organizational capacity for evidence-informed policy-making in Nigeria,^23,24^ and these studies were undertaken in only one (Ebonyi state) of the Nigeria 36 states. There is yet to be wide spread effort to conduct similar studies in other states in Nigeria or at the national level and especially among MNCH stakeholders. The reason for the dearth of this type of studies is partly because there is no sustainable platform for bringing together policy-makers, researchers, and other stakeholders to consider issues around the research to policy and practice interface. Another reason is the scarcity of local funding for evidence-to-policy related research.

Till date there has never been a stakeholders’ engagement event in Nigeria of MNCH policy-makers, researchers, and other stakeholders to discuss evidence-informed policy-making issues regarding MNCH in Nigeria. This type of MNCH stakeholders’ engagement event to consider issues around research to policy and practice interface is very crucial because of the poor state of maternal and child health in Nigeria. The objective of this study was to assess MNCH policy-makers’ capacity for evidence-informed policy-making during a stakeholders’ engagement event as a part of the effort to promote evidence-to-policy-practice for the improvement of maternal and child health in Nigeria. Among the key reasons of convening this event was to use it as a forum of influence on the national stakeholders’ knowledge and capacity with respect to evidence-informed policy-making relevant to MNCH.

## Methods

### Study Design

The study design is a cross-sectional evaluation of MNCH stakeholders’ knowledge as it pertains different dimensions of research to practice. This was undertaken during a national MNCH stakeholders’ engagement event convened under the auspices of the West African Health Organization (WAHO) and the Federal Ministry of Health (FMoH) Nigeria, in October 2015 in Abuja, Nigeria.

### Study Population Selection

A mapping of the stakeholders was undertaken to identify the specific stakeholders that are relevant to the meeting. Senior policy-makers from Nigeria health ministries who are directors, programme managers, and heads of department involved in MNCH programme implementation were identified from the database of FMoH. Other professionals occupying similar positions in selected tertiary educational institutions and national/international non-governmental organizations (NGOs) involved in MNCH programmes in Nigeria were also identified. The criteria for selection of participating organizations and their representatives were as follows: (*i*) Organization/representative must be currently involved in MNCH programmes; (*ii*) Organization/representative must have participated in previously FMoH organized MNCH programmes; and (*iii*) The representative must be a senior official directly involved in MNCH implementation programme in the organization. A total of 92 individuals were invited to the meeting. The profile and representatives of the organizations that participated in the event is shown in Table 1. The participants were drawn from the FMoH Abuja and its associated ministries, departments and agencies (MDAs); others included State Ministry of Health (SMoH); development partners (DPs), civil society organizations (CSOs), NGOs, and the universities/research institutes. Of the 92 participants, 71 (77.17%) were from organizations directly involved in policy-making process (ie, FMoH, MDAs, SMoH, DPs, CSOs, NGOs). The Implementation Research Teams (IRTs) of the IDRC project were also invited and were represented by a member of the team. Invitation letters were sent to participants about three weeks to the meeting, this was followed up with telephone contacts and reminders prior to the meeting.

**Table 1:** Profile and Number of Representatives of the Organizations Participating in the Stakeholders’ Engagement Event

**Type of Organization**	**No. of Representatives (%)**
FMoH headquarters	20 (21.74)
MDAs under FMoH	12 (13.04)
SMoH (17 states and FCT)	18 (19.57)
DPs, CSOs, and NGOs	21 (22.83)
Professional health associations	5 (5.43)
Universities/research institutions	5 (5.43)
WAHO collaborator facilitators	11 (11.96)
Total	92

Abbreviations: FMoH, Federal Ministry of Health; DPs, development partners; NGOs, non-governmental organizations; CSOs, civil society organizations; WAHO, West African Health Organization; SMoH, State Ministry of Health; MDAs, ministries, departments and agencies; FCT, Federal Capital Territory.

### Description of the Data Collection Tools and Process

A questionnaire to assess evidence-informed policy-making knowledge and capacity regarding MNCH was administered to participants from organizations that are directly involved in policy-making process (ie, FMoH, MDAs, SMoH, DPs, CSOs, NGOs) after the completion of an informed consent form. The questionnaire and the informed consent form were approved by the University Research Ethics Committee of Ebonyi State University Nigeria (the institution of the principal author). The approval was based on the agreement that participation in the research was voluntary following informed consent; that participants’ anonymity would be maintained; and that every finding would be treated with utmost confidentiality and for the purpose of this research only. These were adhered to in this study. The questionnaire was designed to assess participants’ knowledge, capacity and organizational process of generation, synthesis and utilization of research evidence in policy-making regarding MNCH.

The questionnaire we developed for this study was principally based on the self-assessment tool produced by the Canadian Health Services Research Foundation (CHSRF) (http://www.cfhi-fcass.ca/Libraries/Documents/SAT-Self-Assessment-Tool.sflb.ashx). The main reason for our choice of the CHSRF self-assessment tool as the basis for the development of our data collection instrument was because of its proven effectiveness. Numerous previous reports have demonstrated that the CHSRF self-assessment tool can help projects evaluate their capacity to use research evidence in the design and delivery of services.^27-31^

### Analysis of the Questionnaire

The data collected via the questionnaires was analyzed using the methods developed at McMaster University, Hamilton, ON, Canada by Johnson and Lavis.^32^ The main parameter measured was participants’ perceptions of their own knowledge/understanding. The analysis is based on mean rating (MNR), median rating (MDR), and range. For instance, the figures represent Likert rating scale of 1–5 points, where 1 point = grossly inadequate; 2 points = inadequate; 3 points = fairly adequate; 4 points = adequate; and 5 points = very adequate.

The range was recorded as the range of values represented by lowest number chosen from the response scale and the highest (eg, 2-5). The mean was calculated as the numbers chosen from the response scale and divide by the total number of responses to the question. While the median was determined by arranging the values chosen from the response scale in ascending order. In terms of analysis, values ranging from 1.00-3.49 points are considered low, whereas values ranging from 3.50-5.00 points considered high. We used 2 decimals for means for a Likert scale measurement because it provided a more accurate representation of respondents view to the questionnaire. None or one decimal will introduce excessive approximation which may not adequately capture the different views of respondents. A number of previous reports used 2 decimals to represent means.^22,23,32^

## Results

### Biodata and Official Designation Attributes

Of the 71 participants from organizations that are directly involved in policy-making process, a total of 40 (56.34%) remained in the meeting until the questionnaire administration period and signed the informed consent form and completed the policy-makers’ questionnaire. Other participants either did not wish to participate in the survey or had left the meeting due to exigencies of duty in their offices. A total of 16 (44.44%) of the respondents were males, and most of the respondents (63.89%) were more that 44 years old. Majority of the respondents (45%) were from the FMoH and its associated MDAs. Most of the respondents (59%) were either directors or chairpersons in their organizations. Most of the respondents have either spent <3 years (37.50%) or 3-5 years (45%) in their present designation. A total of 59% of the respondents have direct influence on policy-making process.

### Knowledge and Application of Information/Communication Technology

The outcome of the participants’ knowledge and application of information/communication technology is presented in Table 2. All the participants indicated that they were computer literate and up to 55.56% had knowledge of basic computer application, however, only 87.50% have a personal computer. All respondents indicated that they use the internet to source for information but only 64.10% use the internet very frequently. The MNR of participants’ knowledge of electronic databases and capacity to identify and obtain relevant research evidence from electronic databases was high and ranged from 3.62-3.68 on the scale of 5 (Table 2).

**Table 2:** Summary of Self-assessment Response of Nigerian Policy-Makers Regarding Knowledge and Application of Information/Communication Technology in MNCH Policy-Making

**Parameter Assessed**	**Outcomes**			
Computer literacy				
Basic computer appreciation	16 (44.44%)			
Basic computer application	20 (55.56%)			
Total	36			
Having a personal computer				
Yes	35 (87.50%)			
No	5 (12.50%)			
Total	40			
Having a computer in your office				
Yes	30 (85.71%)			
No	5 (14.29%)			
Total	35			
Types of operations computer is used for				
Secretarial work	20 (30.30%)			
Data base management	25 (37.88%)			
Data analysis	20 (30.30%)			
Other operations	1 (1.52%)			
Total	66			
Use the internet to source for information				
Very frequently	25 (64.10%)			
Frequently	12 (30.77%)			
Occasionally	2 (5.13%)			
Total	39			
**Individual Knowledge and Capacity**	**Mean**	**Median**	**Range**	**Total**
Of electronic databases where health research evidence can be obtained	3.68	3	2-4	40
To identify and obtain relevant research evidence from electronic databases of health research	3.62	4	2-5	37

Abbreviation: MNCH, maternal, newborn, and child health

### Individual Knowledge of Policy-Making Process

The outcome of the participants’ individual knowledge of policy-making process is presented in Table 3. The MNRs of the participants’ extent of involvement in the policy-making process, level of knowledge of the meaning of policy, understanding of policy context, and knowledge about stakeholders’ and various actors’ involvement in policy-making was relatively high and ranged from 3.82-4.16 on a scale of 5. Also the level of participants understanding of the meaning of a policy brief, a policy dialogue and the role of researchers in policy-making was high ranging from 3.50-3.86 on a scale of 5 (Table 3).

**Table 3:** Summary of Self-assessment Response of Nigerian Policy-Makers Regarding Individual Knowledge of Policy-Making Process in MNCH Policy-Making

**Parameter Assessed**	**Mean**	**Median**	**Range**	**Total**
Extent of involvement in the policy-making process	3.82	4	1-5	39
Knowledge of the meaning of policy	3.97	4	3-5	39
Understanding of policy context	4.16	4	3-5	39
Knowledge about stakeholders’ and various actors’ involvement in policy-making	4.00	4	2-5	40
Understanding of policy-making process	3.92	4	2-5	37
Understanding of the meaning of priority setting/policy agenda in policy-making	3.82	4	2-5	39
Understanding of the meaning of a policy brief	3.61	4	2-5	38
Understanding of what a policy dialogue is	3.50	3	2-5	39
Knowledge on the role of researchers in policy-making	3.86	4	2-5	37

Abbreviation: MNCH, maternal, newborn, and child health.

### Individual Capacity for Use of Evidence

The outcome of the participants’ individual knowledge of policy-making process is presented in Table 4. The MNRs of the participants’ level of understanding on what evidence is in policy-making context, knowledge on the types of evidence that can be used for policy-making, knowledge on the sources of evidence used for policy-making, capacity to identify/select relevant evidence for policy-making, ability to adapt (extract, synthesize, and present) evidence and ability to transform evidence into policy useable form were relatively high. The MNRs ranged from 3.63-4.08 on the scale of 5 (Table 4).

**Table 4:** Summary of Self-assessment Response of Nigerian Policy-Makers Regarding Individual Capacity for Use of Evidence in MNCH Policy-Making

**Parameter Assessed**	**Mean**	**Median**	**Range**	**Total**
Understanding on what evidence is in policy-making context	3.97	4	3-5	39
Knowledge on the types of evidence that can be used for policy-making	3.67	4	2-5	39
Knowledge on the sources of evidence used for policy-making	4.08	4	2-5	40
Capacity to identify/select relevant evidence for policy-making	3.95	4	3-5	38
Ability to adapt (extract, synthesize, and present) evidence used for policy-making	3.68	4	2-5	38
Ability to transform evidence into policy useable form	3.63	4	2-5	38

Abbreviation: MNCH, maternal, newborn, and child health.

### Organizational Geographical Focus and Profile

The outcome of the assessment of participants’ organizational geographical focus and profile is presented in Table 5. The MNRs of the participants’ organization’s capacity/competence to cover their geographical areas of operation were generally low ranging from 3.32-3.38 in terms of manpower, logistics, facilities, and external support. The lowest MNR of 2.66 was recorded in funding. Although most participants indicated the availability of ethical guidelines (76.92%) and ethics/bench marking/best practice unit (64.86%) in their organization, the degree of adherence to guidelines on ethics/bench marking/best practice in their organizations recorded a low MNR of 3.40 on the scale of 5 (Table 5).

**Table 5:** Summary of Self-assessment Response of Nigerian Policy-Makers Regarding Their Organizational Geographical Focus and Profile

**Parameter Assessed**		**Mean**	**Median**	**Range**	**Total**
Organization’s capacity/competence to cover geographical area of operation					
Manpower		3.38	4	1-5	40
Logistics		3.11	3	2-5	35
Funding		2.66	2	1-5	35
Facilities		3.32	3	1-5	34
External support		3.32	4	1-5	38
Accessibility (patronage) of the services provided by organization within the geographical area of operation		3.45	3	2-5	38
Adherence to guidelines on ethics/bench marking/best practice in your organization		3.40	3	2-5	39
Availability of regulatory mechanisms in organization	**Yes (%)**		**No. (%)**		**Total**
Ethical unit in your organization	30 (76.92)		9 (23.08)		39
Document on health research ethics	28 (75.68)		9 (24.32)		37
Document on bench marking/best practice	24 (64.86)		13 (35.14)		37
Organization’s geographical coverage	**Ward (%)**	**LGA (%)**	**State (%)**	**Federal (%)**	**International (%)**
	2 (3.70)	5 (9.26)	17 (31.48)	21 (38.89)	9 (16.67)

### Policy and Policy-Making Process Related to Maternal, Newborn, and Child Health

The outcome of the assessment of policy and policy-making process related to MNCH among the stakeholders’ participants is presented in Table 6. Although majority of the participants indicated the existence of a policy on health research related to MCNH in their organizations (68.42%), stakeholders’ views defined and integrated within a policy on health research related to MNCH (74.29%) and forum or process to coordinate the setting of health research priorities related to MNCH (70.27%), however, the MNRs of the extent of uses of the research done by others and use of research related to MNCH initiated/done by their organizations for policy-making was low ranging from 3.30-3.39 on the scale of 5. The MNRs on the relevance and extent of use of data collected routinely or by survey related to MNCH in their organization for policy-making was high ranging from 3.60-3.97 on a scale of 5 (Table 6).

**Table 6:** Summary of Self-assessment Response of Nigerian Policy-Makers Regarding Policy and Policy-Making Process Related to MNCH

**Parameter Assessed**		**Yes (%)**	**No (%)**	**Total**
Existence of a policy on health research related to MNCH in your organization involving all key stakeholders		26 (68.42)	12 (31.58)	38
Stakeholders’ views defined and integrated within a policy on health research related to MNCH in your organization		26 (74.29)	9 (25.71)	35
Existence of a forum or process to coordinate the setting of health research priorities related to MNCH in your organization		26 (70.27)	11 (29.73)	37
	**Mean**	**Median**	**Range**	**Total**
Extent your organization uses the research done by others related to MNCH	3.30	4	1-5	37
Extent of use of research related to MNCH initiated/done by your organization for policy-making	3.39	4	2-5	36
Extent of use of data collected routinely or by survey related to MNCH by your organization for policy-making	3.60	4	2-5	37
Relevance of evidence related to MNCH used by your organization for policy-making	3.97	4	2-5	33
Policy documents related to MNCH have been made by policy-makers your organization in the last 5 years	2.43	2	1-5	35
Health policies/policy documents related to MNCH have been updated in your organization in the last 5 years	1.81	2	1-4	32

Abbreviation: MNCH, maternal, newborn, and child health.

## Discussion

The outcomes of this study suggest that a stakeholders’ engagement event can serve as an important platform to assess policy-makers’ knowledge and capacity for evidence-informed policy-making and for the promotion of evidence to policy process in general and in MNCH specifically. The meeting afforded the various stakeholders including researchers, policy-makers, DPs, and NGOs opportunity to interact and discuss the constraints and challenges associated with the use of evidence in policy-making in Nigeria. The meeting also served as a kind of training workshop with some capacity building elements during which certain key issues associated with evidence-to-policy link were taught. An earlier report confirmed that meetings like this can enhance the capacity of policy-makers for evidence-informed policy-making.^33^

In a review on research capacity strengthening in the South, Nchinda^34^ noted that stakeholders meeting including workshops on their own have become a useful method of training and calls for greater involvement by policy-makers in developing countries in the entire capacity building process. According to Green and Bennett,^26^ skills in using evidence may be improved through training and development programmes for policy-makers and other policy agents and should be given greater attention in developing countries. Hrynkow and colleagues^35^ added that working to strengthen local expertise and scientific capacity is one of the most effective and lasting ways to affect positive policy change.

Results of the questionnaire assessment indicated that all participants were computer literate and make use of the internet to source information but only 64.10% of the stakeholders noted that they use the internet very frequently. This finding suggests that most of the participants are aware of the availability of evidence which can be accessed via the internet. Furthermore, result also showed that knowledge of electronic databases and capacity to identify and obtain relevant research evidence from electronic databases was very high and ranged from 3.62-4.60 on the scale of 5. This is a positive outcome and a catalyst in the promotion of evidence to policy process.

Available studies have indicated that information and communication technologies (ICTs) have the potential to make a major contribution to improving access and quality of services while containing costs,^36^ and could provide fast, efficient and relatively cheap access to information leading to dramatic improvements in access to advice and care.^37^ Reports by the United Nations Educational, Scientific and Cultural Organization (UNESCO)^38^ and WHO^39^ noted that we are living in what are increasingly referred to as “knowledge societies” which are able to harness the huge amount of information that modern technology such as computers and the Internet allow us to manipulate, store, transmit and share. According to Green and Bennett,^26^ the skill, therefore, lies in turning all this information into knowledge; and the great challenge is to then use that knowledge-to put it into practice.

Concerning individual knowledge of the policy-making process and capacity for use of evidence, result showed that the participants appeared to have a considerable knowledge of what the policy-making process entails including knowledge of the meaning of policy, understanding of policy context, and knowledge about stakeholders’ and various actors’ involvement in policy-making as indicated by the MNRs which ranged from 3.63-4.60. This result was not unexpected as there is an increasing awareness of the policy-making process by stakeholders in the health sector worldwide.

Available reports have indicated that there is an increasing recognition world wide of the importance and necessity of the use of more rigorous processes to ensure that healthcare recommendations are informed by the best available research evidence.^1,2^ At the same time, the search for strategies to get research findings into policy and practice has gained momentum and the global literature has called for further exploration in the area of research to policy.^40^ The need and ways to engage decision-makers into health research covers an extensive scientific field. Therefore, engaging decision-makers in specific areas of health research, has been advocated as one of the solutions to address this challenge, for example by the use of surveys of decision-makers.^41^

In terms of the participants organizational geographical focus and profile the MNRs of the participants’ organization’s capacity/competence to cover their geographical areas of operation were generally low ranging from 3.32-3.38 in terms of manpower, logistics, facilities, and external support. The lowest MNR of 2.66 was recorded in funding. This clearly suggests that there exist significant challenges in the infrastructure, manpower, and funding in the health policy-making process and implementation in Nigeria. Although the problem of accessibility is another important factor because even when evidence is available, policy-makers may have problems obtaining it. Lack of funds for sustained subscription to evidence sources such as databases and journals may hamper access.^22^ In addition, some of the policy-makers particularly at the regional and local government levels may not have basic information technology skill to access research evidence relevant for policy-making.^22,24^

Interestingly, the participants noted that their organizations generally had a poor attitude towards the use of the research done by others (eg, researchers) and that there was little interest towards the updating of MNCH policy documents. This development is worrisome and poses a critical challenge to the evidence-informed policy-making process regarding MNCH in Nigeria, because inadequate organizational capacity and commitment towards evidence-informed policy-making can incapacitate even the highly knowledgeable and skillful policy-maker.^22^ Therefore, any planned intervention should place emphasis on strategies that will improve organizational capacity for adapting research evidence in policy-making.^23^ Another possible reason leading to poor organizational attitude and the limited usability of existing research evidence by policy-makers is the fact that policy-makers’ needs do not drive research.^42,43^ Unfortunately, academic researchers generally follow their own interests when choosing what studies to conduct or tailor them to specific requests for grants. Similarly, the synthesis of existing research in the form of systematic reviews is driven by the researchers’ particular interests and not necessarily based on local policy-makers information needs.^3,44^ This is why training of policy-makers and initiating strategies to enhance organizational capacity for the application of research evidence are a vital aspect of the interventions to improve evidence to policy process in MNCH in low-income settings.

## The Limitation of Study

This study has three major limitations. First, our use of only a quantitative cross-sectional approach fails to provide adequate information on the context of the situation where the studied phenomenon occurs. This limitation was also reported in a previous similar study in Nigeria.^23^ We, therefore, recommend for future studies the inclusion of descriptive study strategy such as a qualitative or causal relationship based on a longitudinal study technique. The second limitation of this study is the weakness of the self-assessment technique which we used to evaluate the study outcome. Even though there are some merits with this method, Deans and Ademokun,^27^ highlighting the weakness of this technique noted that being able to critically recognize and understand one’s own gap in skills and knowledge is a difficult process which takes guided thought. Furthermore, Haahr and colleagues^45^ described self-assessments as subject to self-esteem bias, may be unreliable, and are difficult to validate. Our inability to include inferential statistics is another limitation to this study. Inferential statistics could have added to the strength of this paper, however, the descriptive nature of the paper is also very valuable and has to large extent generated information that will enable the development of capacity improvement intervention. We recommend the inclusion of inferential statistics in future studies. These weaknesses notwithstanding, the findings from this present study could serve as a pointer to the various areas of capacity constraints that require urgent intervention to improve MNCH policy-making process.

## Conclusion

As efforts are made towards the development of more effective policies that will improve MNCH outcomes in low-income settings, the enhancement of policy-makers’ capacities for evidence-informed policy-making becomes very imperative. Assessing policy-makers’ capacity for evidence-to-policy link is, therefore, vital in order to identify the specific areas of capacity constraints requiring urgent attention. This present study has provided valuable scientific information that will aid in the development of intervention strategies that will improve the policy-making skill and competence of MNCH decision-makers in Nigeria. Similar assessment is highly recommended to other resource poor settings.

## Acknowledgments

Authors are grateful to the policy-makers, researchers, and other stakeholders who participated in this study. This study was one of the outcomes of the “Moving Maternal, Neonatal and Child Health Evidence into Policy in West Africa” ​​(MEP) project undertaken by WAHO funded by International Development Research Centre (IDRC), Ottawa, ON, Canada (Reference: IDRC 107892_001).

## Ethical issues

The study was approved by the University Research Ethics Committee of Ebonyi State University Nigeria, Abakaliki, Nigeria.

## Competing interests

Authors declare that they have no competing interests.

## Authors’ contributions

Study conception and design by all authors. Data acquisition, analysis and interpretation by CJU, IS, NK, and PO. Drafting of the manuscript by CJU. Critical revision of the manuscript for important intellectual content by IS and PO. Obtaining funding by IS, NK, VL, and EJ. Administrative, technical, and material support by all authors.

## Authors’ affiliations

^1^Knowledge Translation Platform, African Institute for Health Policy and Health Systems Studies, Ebonyi State University, Abakaliki, Nigeria. ^2^Organisation Ouest Africaine de la Santé, Bobo-Dioulasso, Burkina Faso. ^3^Hopital Central Yaounde, Yaoundé, Cameroon.

## Key messages

**Implications for policy makers**

- An assessment of policy-makers’ capacity constraints regarding evidence-informed policy-making is an important first step towards the implementation of evidence-based interventions to improve the evidence-to-policy link.
- Any planned intervention should place emphasis on strategies that will improve organizational capacity for adapting research evidence in policy-making.
- Training of policy-makers and initiating strategies to enhance organizational capacity for the application of research evidence are a vital aspect of the interventions to improve evidence to policy process in maternal, newborn, and child health (MNCH) in low-income settings.

**Implications for public**

There is no proper uptake of research evidence into policy-making by decision-makers in low- and middle-income countries, and this may be due to individual and organizational capacity constraints regarding evidence to policy link. The identification of the specific capacity challenges of policy-makers and their organization is the first step for developing effective health policy.
